# Clinician Bias and Patient Experience in Cancer Genetic Counseling

**DOI:** 10.1001/jamanetworkopen.2026.39990

**Published:** 2026-10-26

**Authors:** Nao Hagiwara, Yongyun Shin, Sabina Yeasmin, Erin P. Carmany, Susan Eggly, Shown C. T. Jones, Nadia Harika, Anaya Gorham, John Quillin

**Affiliations:** 1 Department of Public Health Sciences, University of Virginia, Charlottesville; 2 Department of Biostatistics, Virginia Commonwealth University, Richmond; 3 Department of Statistics and Data Science, Jahangirnagar University, Savar, Dhaka, Bangladesh; 4 Center for Molecular Medicine and Genetics, Karmanos Cancer Institute, Wayne State University, Detroit, Michigan; 5 Department of Oncology, Karmanos Cancer Institute, Wayne State University, Detroit, Michigan; 6 Department of Psychology, Virginia Commonwealth University, Richmond; 7 Department of Pediatrics, Virginia Commonwealth University, Richmond

## Abstract

**Importance:**

Genetic counseling helps reduce cancer burden; however, disparities exist in genetic counseling.

**Objective:**

To examine associations between genetic counselor (GC) biases (implicit and/or explicit prejudice and stereotyping) and patient-centered outcomes in cancer genetic counseling.

**Design, Setting, and Participants:**

This is a cohort study of patient-clinician communication during cancer genetic counseling focusing on hereditary cancer risk assessment. GCs and patients completed baseline surveys, video and/or audio-recorded sessions, and postencounter surveys. Data were collected at 2 comprehensive cancer centers in the South and Midwest, from August 3, 2023, to July 30, 2025.

**Exposure:**

At baseline, GCs completed measures of implicit prejudice (Race Implicit Association Test), implicit stereotyping (Medical Cooperativeness Implicit Association Test), explicit prejudice (feeling thermometer), and explicit stereotyping (general and medical-context stereotype subscales).

**Main Outcomes and Measures:**

Patients reported postencounter therapeutic alliance, satisfaction, and decisional conflicts. Differences between Black and White patients were evaluated using Welch *t *test for continuous variables and χ^2^ or Fisher exact test for categorical variables. Hypothesis testing was conducted using linear mixed-effects models with GC-specific random effects and intracluster correlation coefficients.

**Results:**

The current analysis included 185 cases with complete clinician baseline, patient baseline, and patient postencounter data. The 185 patients (mean [SD] age, 50.1 [14.0] years; 169 female [91.4%]; 16 male [8.6%]; 83 Black [44.9%]; 102 White [55.1%]) saw 1 of the 15 clinicians (mean [SD] age, 32.2 [9.5] years; 8 certified [53.3%]; 7 trainees [46.7%]). Among Black patients (mean [SD] age, 50.1 [13.6] years; 80 female [96.4%]; 3 male [3.5%]), stronger GC endorsement of explicit medical-context stereotypes favoring White over Black patients was significantly associated with poorer therapeutic alliance (*b *[SE], −1.40 [0.37] to −1.54 [0.46]), lower satisfaction (*b *[SE], −0.74 [0.27]), and greater decisional conflict (*b *[SE], −0.85 [0.27] to −0.91 [0.25]), after covariate adjustment. Among White patients (mean [SD] age, 50.1 [14.4] years; 89 [87.3%] female; 13 [12.7%] male), no biases were significantly associated with outcomes after adjustment (*b *[SE], −0.14 [0.49] to −0.48 [0.49]). Explicit medical-context stereotyping was the only factor significantly and consistently associated with racial disparities in patient-centered outcomes (*b *[SE], −1.35 [0.49] to −1.93 [0.61]).

**Conclusions and Relevance:**

In this cohort study of cancer genetic counseling, stronger endorsement of medical-context stereotypes favoring White over Black patients was significantly associated with poorer patient-centered outcomes among Black patients. Explicit medical-context stereotypes may contribute to racial disparities in genetic counseling. Interventions should address explicit stereotyping and its role in clinical communication.

## Key Points

**Question:**

How are different forms of clinician bias associated with racial disparities in patient-centered outcomes during cancer genetic counseling?

**Findings:**

This cohort study of survey data from a study of patient-clinician communication during cancer genetic counseling sessions for 185 patients and 15 clinicians revealed that, among Black, but not White, patients, clinicians’ explicit medical-context stereotyping favoring White over Black patients was significantly associated with poorer therapeutic alliance, lower satisfaction, and greater decisional conflict.

**Meaning:**

These findings suggest that bias training should extend beyond implicit bias to directly address explicit stereotyping and its role in clinical communication.

## Teaser

This cohort study examines associations between genetic counselor biases (implicit or explicit prejudice and stereotyping) and patient-centered outcomes in cancer genetic counseling.

## Introduction

Hereditary cancer syndrome prevalence is similar across races in the US.^1,2,3^ However, Black individuals face higher risks of several cancers (eg, prostate, colorectal, and pancreatic)^4,5,6^ and have the highest overall cancer mortality.^7^ Although factors contributing to these disparities span from patient characteristics (eg, socioeconomic status and health literacy)^8,9,10^ to societal determinants (eg, geographic inequities and access to care),^9,10,11,12^ cancer genetic counselors (GCs) have the potential to mitigate disparities.

Genetic counseling is one key entry point into cancer care, helping reduce cancer burden. Cancer GCs assess hereditary cancer risks; address medical, psychological, and familial concerns; and provide guidance on prevention, screening, and treatment options.^13,14^ Importantly, GCs are trained to provide patient-centered care by building rapport, eliciting and understanding patient perspectives, supporting informed decision-making, and tailoring communication to patients’ cultural, emotional, and cognitive needs.^15,16,17,18,19^ These skills align closely with the core communication skills outlined by the American Society of Clinical Oncology.^20^

However, disparities exist within the genetic counseling process itself.^21^ Compared with White individuals, individuals of other races, including Black individuals, have reported lower satisfaction and a reduced sense of support during genetic counseling^22^ and were also less likely to pursue genetic testing.^23^ As documented in other clinical areas, one contributor to these disparities may be clinician bias.^24,25,26,27^ Bias is a multidimensional construct including prejudice (negative feelings toward a group and its members) and stereotypes (preconceived beliefs about a group or members), which can manifest as discrimination (harmful actions against a group or members).^28,29^ These components can operate at implicit (automatic or spontaneous) and explicit (deliberate or effortful) levels.^28,29^

Within genetic counseling, implicit prejudice favoring White over Black individuals has been associated with less individualized communication^30^ and less communication of emotional^31^ and cognitive information.^32^ Genetic counseling trainees with higher levels of implicit prejudice used more frequent back-channeling, had longer sessions, and used fewer facilitative statements when interacting with White (vs Black) standardized patients.^33^ Finally, GCs (both certified and trainees) hold both implicit prejudice and implicit medical cooperativeness stereotypes that disfavor Black people.^34^ GCs also explicitly endorsed stereotypes that Black people are mistrustful of the health care system.^34^ Collectively, these findings indicate that GCs, like other health care professionals, may exhibit bias.^35,36^

However, prior research has several major gaps. First, most studies focused primarily on implicit prejudice, overlooking other forms of bias, even though evidence suggests that different biases manifest in distinct health care aspects.^30,31,32,33^ Specifically, higher levels of implicit prejudice are associated with poorer quality of patient-clinician communication, and stronger endorsement of negative stereotypes about Black individuals (ie, explicit stereotypes) is associated with suboptimal clinical recommendations.^25^ There is also emerging evidence that these forms of bias may not operate in isolation. For example, clinicians with an aversive racism profile (ie, high implicit but low explicit prejudice) tended to have shorter interactions with Black patients^37^ and to be perceived least favorably by Black patients compared with any other clinicians (ie, high implicit and explicit prejudice, low implicit and explicit prejudice, low implicit but high explicit prejudice).^38^ Second, although one study examined all 4 forms of bias among GCs, it did not examine their associations with outcomes.^34^ Third, existing studies linking GC bias to patient-centered outcomes have relied on simulations with standardized patients,^30,31,32,33^ which may not reflect real patient experiences.^39,40^ Finally, prior work has largely emphasized communication processes^30,31,32,33^ rather than patient-reported outcomes that are meaningful to patients and more directly reflect genetic counseling goals (ie, patient-centered outcomes), such as improving patient decision-making about genetic testing.

The present study addressed these gaps by examining associations between multiple forms of GC bias and patient-centered outcomes among Black and White patients. We hypothesized that higher (vs lower) levels of implicit prejudice would be associated with less positive reactions from Black patients but more positive reactions from White patients (hypothesis 1); that GCs with an aversive racism profile would elicit the least positive reactions from Black patients, but not from White patients (hypothesis 2); and that stronger (vs weaker) endorsement of medical-context stereotype favoring White over Black individuals would be associated with less positive reactions from Black patients and more positive reactions from White patients (hypothesis 3). We also explored which form of bias was most strongly and reliably associated with patient-centered outcomes (question 1). eFigure 1 in Supplement 1 provides a visual overview of the hypotheses.

## Methods

This cohort study analyzed survey data examining patient-clinician communication during cancer genetic counseling sessions that focused on assessment of and counseling for hereditary cancer risk.^41^ A convenience sample of 15 GCs and 245 patients was enrolled in the parent study at 2 comprehensive cancer centers in the South and Midwest, between August 3, 2023, and July 30, 2025 (eAppendix 1 and eFigure 2 in Supplement 1). Eligible clinicians were American Board of Genetic Counseling–certified counselors or genetic counseling trainees in at least their second cancer genetics rotation. Eligible patients were (1) self-identified as Black or African American or White, (2) aged at least 18 years, (3) scheduled for a hereditary cancer–focused counseling session with a participating clinician, and (4) able to read English at a sixth-grade level. Detailed information about the screening and recruitment procedure is provided in eAppendix 2 in Supplement 1. GCs and patients completed baseline surveys, video and/or audio-recorded sessions, and postencounter surveys (detailed measure descriptions are available elsewhere).^41^ The parent study was approved by the Virginia Commonwealth University institutional review board, and all participants provided written informed consent. This study is reported according to the Strengthening the Reporting of Observational Studies in Epidemiology (STROBE) reporting guidelines for cohort studies.

### Measures

#### Explanatory Factors (Baseline)

Patient race was assessed with self-report. Clinician implicit prejudice was assessed using the Race Implicit Association Test,^42,43^ which measures automatic associations between racial groups (Black vs White) and evaluations (good vs bad). Scores were computed as *D* scores (range −2.0 to 2.0; split-half reliability with Spearman-Brown correction α = 0.78),^44^ with positive values bias favoring White and/or disfavoring Black individuals.

Clinician implicit stereotyping was assessed with the Medical Cooperativeness Implicit Association Test, replacing “good/bad” with “medically cooperative/uncooperative.”^45,46^ *D* scores were calculated as above, with positive values indicating stronger associations of White individuals with cooperativeness.

Clinician explicit prejudice was measured using a feeling thermometer, in which participants rated warmth toward racial groups from 0 (very cold) to 100 (very warm).^47^ Composite scores subtract ratings for Black individuals from those of White individuals; positive values indicate bias favoring White individuals.

Clinician explicit stereotyping was assessed using 2 subscales: general (32 items assessing general racial stereotypes, such as intelligence, laziness, and socioeconomic status) and medical-context (34 items assessing stereotypes specific to health care settings, such as medical compliance, health literacy, and motivation).^34^ Items were rated from 1 (very untrue) to 6 (very true). Composite scores were calculated by averaging responses (after necessary reverse-coding) and subtracting Black from White ratings; positive values indicate more favorable stereotypes of White individuals.

#### Outcomes (Postencounter)

Patient-perceived therapeutic alliance (perceived relationship with their GC) was assessed using the Working Alliance Inventory–Short Revised, client version with 3 subscales: tasks for achieving the goals of the genetic counseling session (α = 0.83), bond between the clinician and patient (α = 0.88), and the goals of the genetic counseling session (α = 0.83).^48^ Patient satisfaction with the clinician and session was assessed using the 6-item Genetic Counseling Satisfaction Scale (α = 0.88).^49^

 Patient decisional conflict was measured with a 16-item Decisional Conflict Scale,^50^ adapted for hereditary cancer testing, with 3 subscales: decision uncertainty (α = 0.79), factors contributing to uncertainty (α = 0.91), and perceived effective decision-making (α = 0.93). Because each item was worded positively, higher numbers indicate less decisional conflict.

#### Covariates

Demographics included patient age, sexual and gender minority status, ethnicity, clinician age, gender, sexual orientation, and race. Clinician professional characteristics included status (certified vs trainee) and site.

Patients’ baseline perceived racial discrimination was assessed using 2 measures. The Brief Perceived Ethnic Discrimination Questionnaire–Community Version captured lifetime and everyday discrimination across domains (α = 0.93),^51^ and a second measure assessed personal (5 items; α = 0.94) and group-level (3 items; α = 0.95) discrimination.^52^ Patients’ baseline decisional conflict was assessed with the same adapted Decisional Conflict Scale.

### Statistical Analysis

Key variables were summarized using descriptive statistics, and pairwise correlations were examined to assess marginal bivariate linear associations. Differences between Black and White patients were evaluated using Welch *t* test for continuous variables and χ^2^ or Fisher exact test for categorical variables. Only outcomes that showed significant associations with explanatory factors in these analyses were further examined using linear mixed-effects models with GC-specific random effects and intracluster correlation coefficients to quantify the degree of dependence among patients clustered within the same clinician. To identify the strongest and most reliable bias-outcome associations, we fitted linear mixed-effects models regressing each therapeutic alliance subscale on 5 clinician bias variables and their interactions with patient race. Statistical significance was defined at 2-sided *P* < .05. Data analysis was performed with R statistical software version 4.6.1 (R Project for Statistical Computing).

## Results

### Enrolled Participants

The current analysis included 185 cases with complete clinician baseline, patient baseline, and patient postencounter data, resulting in 185 patients (mean [SD] age, 50.1 [14.0] years; 169 female [91.4%]; 16 male [8.6%]; 83 Black [44.9%]; 102 White [55.1%]) who saw 1 of the 15 clinicians (mean [SD] age, 32.2 [9.5] years; 8 certified [53.3%]; 7 trainees [46.7%]). Of the Black patients, (mean [SD] age, 50.1 [13.6] years), 80 (96.4%) were female, and 3 (3.5%) were male. Of the White patients (mean [SD] age, 50.1 [14.4] years), 89 (87.3%) were female, and 13 (12.7%) were male (Table 1).

**Table 1.** Participant Characteristics

Characteristic	All	Subgroup 1	Subgroup 2
**Genetic counselor, mean (SD)^a^**	**All (N = 15)**	**Certified (n = 8)**	**Trainee (n = 7)**
Age, y	32.20 (9.45)	37.50 (10.03)	26.14 (3.29)
Time in practice, y	NA	13.38 (9.78)	NA
Implicit prejudice score	−0.11 (0.58)	−0.16 (0.63)	−0.05 (0.57)
Implicit stereotyping score	−0.01 (0.37)	0.08 (0.33)	−0.11 (0.42)
Explicit prejudice score	0.07 (12.94)	3.88 (10.56)	−4.29 (14.80)
Explicit stereotyping (general) score	−0.70 (0.58)^b^	−0.49 (0.67)	−0.93 (0.39)
Explicit stereotyping (medical context) score	0.03 (0.30)	0.02 (0.23)	0.04 (0.38)
**Patients, No. (%)**	**All (N = 185)**	**Black (n = 83)**	**White (n = 102)**
Age, mean (SD), y	50.11 (14.01)	50.12 (13.56)	50.11 (14.43)
Sex			
Female	169 (91.4)	80 (96.4)	89 (87.3)
Male	16 (8.6)	3 (3.6)	13 (12.7)
Gender			
Woman	166 (89.7)	78 (94.0)	88 (86.3)
Man	17 (9.2)	4 (4.8)	13 (12.7)
Nonbinary	1 (0.5)	0	1 (1.0)
Multiple selections	1 (0.5)	1 (1.2)	0
Sexual and gender minority status			
Yes	18 (9.2)	8 (9.6)	9 (8.8)
No	168 (90.8)	75 (90.4)	93 (91.2)
Education			
No school	1 (0.5)	0	1 (1.0)
12th Grade or less	8 (4.3)	5 (6.0)	3 (2.9)
High school graduate	31 (16.8)	16 (19.3)	15 (14.7)
Trade, vocation, or certificate program	6 (3.2)	4 (4.8)	2 (2.0)
Some college	21 (11.4)	13 (15.7)	8 (7.8)
Associate’s degree	22 (11.9)	12 (14.5)	10 (9.8)
Bachelor’s degree	45 (24.3)	18 (21.7)	27 (26.5)
Postgraduate degree	51 (27.6)	15 (18.1)	36 (35.3)
Annual household income, $ (missing = 4)			
<25 000	31 (16.8)	27 (32.5)	4 (3.9)
25 000-49 999	44 (23.8)	26 (31.3)	18 (17.6)
50 000-74 999	25 (13.5)	10 (12.0)	15 (14.7)
75 000-99 999	13 (8.1)	3 (3.6)	12 (11.8)
100 000-149 999	33 (17.8)	11 (13.3)	22 (21.6)
150 000-199 999	12 (6.5)	1 (1.2)	11 (10.8)
≥200 000	21 (11.4)	3 (3.6)	18 (17.6)

Abbreviation: NA, not applicable.

^a^ Although genetic counselors’ gender, sexual orientation, and race were assessed and included in hypothesis testing, these variables are not reported to minimize the risk of participant reidentification.

^b^ Indicates that the overall mean was significantly different from 0 with *P* < .05 (ie, differential preference based on race).

### Descriptive Statistics

GCs reported, on average, statistically significant levels of explicit general stereotyping favoring Black patients over White patients (*t*_14_ = 4.61; Cohen *d* = 0.58). Other biases did not differ from zero (*t*_14_ = 0.20 to 0.74). Black patients reported significantly less favorable outcomes than White patients on 5 of 7 assessed outcomes (*t*_183_ = 2.02 to 2.31; Cohen *d* = 0.51 to 0.78) (Table 2).

**Table 2.** Pairwise Correlations Between Explanatory Factors and Outcomes, and Outcome by Patient Race

Explanatory factors	Correlation coefficients													
	WAI tasks		WAI bond		WAI goals		GCSS		DCS decision		DCS factors		DCS effective decision-making	
	Black	White	Black	White	Black	White	Black	White	Black	White	Black	White	Black	White
Implicit prejudice	−0.04	0.10	−0.06	0.30^a^	−0.04	0.15	0.08	0.07	0.05	0.12	0.04	−0.01	0.11	0.06
Implicit stereotyping	0.03	0.09	0.01	0.24^a^	0.02	0.13	0.18	0.09	0.10	−0.02	0.13	−0.09	0.17	−0.07
Explicit prejudice	0.08	0.12	0.09	0.06	0.08	0.07	0.16	0.14	0.11	−0.001	0.19	0.10	0.18	0.04
Explicit stereotyping (general)	0.18	−0.02	0.08	0.03	0.15	−0.14	0.11	−0.03	−0.004	0.11	0.12	−0.01	0.12	0.13
Explicit stereotyping (medical context)	−0.39^b^	−0.05	−0.32^c^	−0.02	−0.35^c^	−0.01	−0.24^a^	−0.13	−0.09	−0.11	−0.28^a^	−0.05	−0.24^a^	−0.06
Black and White patient comparison, mean (SD), score	4.25 (−0.70)^d^	4.45 (−0.50)	4.07 (−0.85)	4.23 (−0.71)	4.18 (−0.64)	4.29 (−0.62)	4.26 (−0.58)^d^	4.44 (−0.49)	4.13 (−0.72)^d^	4.36 (−0.69)	4.27 (−0.53)^d^	4.42 (−0.49)	4.39 (−0.55)^d^	4.56 (−0.48)

Abbreviations: DCS, Decisional Conflict Scale; GCSS, Genetic Counseling Satisfaction Scale; WAI, Working Alliance Inventory.

^a^ *P* < .05.

^b^ *P* < .001.

^c^ *P* < .01.

^d^ Indicates a significant difference (*P* < .05) between the mean values for Black and White patients. The possible range for the composite scores of WAI, GCSS, and DCS is 1 to 5.

Among Black patients, GCs’ stronger endorsement of medical-context stereotypes favoring White over Black patients was significantly associated with lower therapeutic alliance across all 3 subscales (*r* = −0.32 to −0.39), lower satisfaction (*r* = −0.24), and higher decision conflict on 2 subscales, factors contributing to uncertainty (*r* = −0.28) and perceived effectiveness of decision-making (*r* = −0.24). Among White patients, GCs’ implicit prejudice (*r* = 0.30) and implicit stereotyping (*r* = 0.24) were significantly associated with the therapeutic alliance goals subscale.

### Hypothesis Testing

After accounting for relevant covariates and data nonindependence using linear mixed-effects models (Table 3), the unadjusted bivariate correlation between implicit prejudice and the bond (*b* [SE], 0.23 [0.32]) subscale of perceived therapeutic alliance were no longer statistically significant. Additionally, the interaction between implicit and explicit prejudice was not significant in any model (*b* [SE], 0.04 [0.08] to 0.22 [0.16]). Fitted linear mixed-effects models showed that, among Black patients, GCs’ explicit medical-context stereotyping remained significantly and negatively associated with poorer therapeutic alliance (*b *[SE], −1.40 [0.37] to −1.54 [0.46]), lower satisfaction (*b *[SE], −0.74 [0.27]), and greater decisional conflict (*b *[SE], −0.85 [0.27] to −0.91 [0.25]), after covariate adjustment. Among White patients, no biases were significantly associated with outcomes after adjustment (*b *[SE], −0.14 [0.49] to −0.48 [0.49]) (Table 4).

**Table 3.** Linear Mixed-Effects Models Testing Hypotheses 1 and 2^a^

Variable	*b* (SE)				
	Hypothesis 1: WAI bond	Hypothesis 2			
		WAI tasks	WAI bond	WAI goals	GCSS
Intercept	3.88 (0.99)^b^	2.65 (2.33)	2.33 (2.62)	1.41 (2.39)	4.70 (1.10)^c^
Race	0.22 (0.16)	0.08 (0.13)	0.19 (0.17)	0.28 (0.14)^b^	0.10 (0.11)
Implicit prejudice	0.23 (0.32)	0.56 (0.74)	0.48 (0.81)	0.75 (0.75)	0.19 (0.28)
Explicit prejudice	NA	−0.05 (0.05)	−0.03 (0.05)	−0.07 (0.05)	−0.00 (0.02)
Race by implicit prejudice	−0.47 (0.18)^b^	−0.09 (0.22)	−0.15 (0.28)	−0.18 (0.23)	0.12 (0.19)
Race by explicit prejudice	NA	−0.00 (0.02)	−0.01 (0.02)	0.00 (0.02)	−0.01 (0.01)
Implicit prejudice by explicit prejudice	NA	0.16 (0.16)	0.05 (0.18)	0.22 (0.16)	0.04 (0.08)
Race by implicit prejudice by explicit prejudice	NA	0.02 (0.06)	0.12 (0.08)	−0.00 (0.06)	0.03 (0.06)
Between-group variance, squared units of the corresponding outcome measure	0.04	0.21	0.24	0.22	0.01
Within-group variance, squared units of the corresponding outcome measure	0.50	0.30	0.49	0.33	0.24
Intracluster correlation	0.08	0.42	0.33	0.40	0.03

Abbreviations: GCSS, Genetic Counseling Satisfaction Scale; NA, not applicable; WAI, Working Alliance Inventory.

^a^ Race was dummy-coded with White patients as the reference group. The models controlled for both patient characteristics (age, sexual and gender minority status, ethnicity, and baseline perceived racial discrimination), clinician characteristics (age, gender, sexual orientation, and race), and clinician professional characteristics (status and site).

^b^ *P* < .05.

^c^ *P* < .01.

**Table 4.** Linear Mixed-Effects Models Testing Hypothesis 3^a^

Variable	*b* (SE)					
	WAI tasks	WAI bond	WAI goals	GCSS	DCS factors	DCS effective decision-making
Intercept	3.98 (0.77)^b^	2.93 (0.98)^c^	2.32 (0.91)^c^	4.54 (0.62)^b^	−0.86 (0.55)	4.64 (0.60)^b^
Race	0.10 (0.12)	0.28 (0.16)	0.28 (0.13)^d^	0.13 (0.11)	0.12 (0.10)	−0.01 (0.11)
Explicit stereotyping (medical context)	−0.31 (0.39)	−0.14 (0.49)	−0.48 (0.49)	−0.23 (0.29)	−0.34 (0.26)	−0.31 (0.28)
Race by explicit stereotyping (medical context)	−1.09 (0.37)^c^	−1.27 (0.49)^d^	−1.06 (0.39)^c^	−0.51 (0.33)	−0.57 (0.30)	−0.54 (0.32)
Effect sizes for Black patients						
Explicit stereotyping (medical context)	−1.40 (0.37)^b^	−1.41 (0.46)^c^	−1.54 (0.46)^b^	−0.74 (0.27)^d^	−0.91 (0.25)^d^	−0.85 (0.27)^d^
Between-group variance, squared units of the corresponding outcome measure	0.02	0.02	0.05	0.00	0.00	0.00
Within-group variance, squared units of the corresponding outcome measure	0.29	0.49	0.31	0.23	0.18	0.21
Intracluster correlation	0.06	0.04	0.14	NA	0.00	0.01

Abbreviations: DCS, Decisional Conflict Scale; GCSS, Genetic Counseling Satisfaction Scale; NA, not applicable; WAI, Working Alliance Inventory.

^a^ Race was dummy-coded with White patients as the reference group. The models controlled for both patient characteristics (age, sexual and gender minority status, ethnicity, baseline perceived racial discrimination, and baseline decisional conflicts), clinician characteristics (age, gender, sexual orientation, and race), and clinician professional characteristics (status and site).

^b^ *P* < .001.

^c^ *P* < .01.

^d^ *P* < .05.

### Exploratory Analysis

The interaction between race and explicit medical-context stereotyping exhibited the strongest associations across all 3 outcomes. All effects were in the negative direction and reached statistical significance (*b* [SE], −1.35 [0.51] to −1.93 [0.61]) (Table 5).

**Table 5.** Linear Mixed-Effects Models Exploring Research Questions^a^

Variable	*b* (SE)		
	WAI task	WAI goals	WAI bond
Intercept	4.00 (0.70)^b^	3.27 (0.81)^b^	2.82 (0.91)^c^
Race	0.09 (0.12)	0.26 (0.13)^c^	0.28 (0.16)
Implicit prejudice	0.11 (0.27)	0.11 (0.35)	0.35 (0.37)
Implicit stereotyping	−0.44 (0.47)	−0.30 (0.57)	−0.45 (0.62)
Explicit prejudice	0.00 (0.01)	−0.00 (0.01)	−0.00 (0.01)
Explicit stereotyping (general)	0.07 (0.19)	−0.04 (0.25)	0.17 (0.26)
Explicit stereotyping (medical context)	0.02 (0.53)	−0.21 (0.65)	0.37 (0.70)
Race by implicit prejudice	0.02 (0.22)	0.05 (0.23)	−0.15 (0.28)
Race by implicit stereotyping	−0.40 (0.44)	−0.63 (0.46)	−0.67 (0.56)
Race by explicit prejudice	−0.01 (0.01)	−0.01 (0.01)	−0.00 (0.01)
Race by explicit stereotyping (general)	−0.10 (0.16)	0.01 (0.17)	−0.28 (0.21)
Race by explicit stereotyping (medical context)	−1.49 (0.47)^b^	−1.35 (0.51)^b^	−1.93 (0.61)^b^
Between-group variance, squared units of the corresponding outcome measure	0.03	0.07	0.05
Within-group variance, squared units of the corresponding outcome measure	0.28	0.31	0.47
Intracluster correlation	0.09	0.19	0.10

Abbreviation: WAI, Working Alliance Inventory.

^a^ Race was dummy-coded with White patients as the reference group. The models controlled for both patient characteristics (age, sexual and gender minority status, ethnicity, and baseline perceived racial discrimination), clinician characteristics (age, gender, sexual orientation, and race), and clinician professional characteristics (status and site).

^b^ *P* < .01.

^c^ *P* < .05.

## Discussion

The present study did not support hypotheses 1 or 2, which proposed that implicit prejudice or its interaction with explicit prejudice (ie, aversive racism profile) would be associated with poorer patient-centered outcomes among Black patients and better outcomes among White patients. However, hypothesis 3 was partially supported: explicit medical-context stereotyping was significantly associated with worse patient-centered outcomes among Black patients, but not among White patients. Exploratory analyses further indicated that explicit medical-context stereotyping showed the most robust associations with therapeutic alliance across all subscales.

The lack of consistent associations between GC implicit prejudice and patient-centered outcomes contrasts with findings commonly reported among non-GC clinicians.^24,25,26,27^ There are at least 4 plausible explanations for these results. First, GCs receive extensive training in rapport-building and communication skills, with a strong emphasis on eliciting and responding to patients’ concerns and needs.^15,16,17,18,19^ This training may help GCs better mitigate the consequences of implicit prejudice in clinical interactions compared with other specialties. Second, cancer genetic counseling sessions for new adult patients typically last approximately 45 minutes,^53^ significantly longer than many other clinical encounters.^54^ This extended time frame may provide GCs with a greater opportunity for individuation (ie, perceiving and treating patients as unique individuals rather than as members of a social category), which can reduce biased judgments, attitudes, and behaviors based on category-based processing.^55,56,57^ Importantly, individuation is also 1 of the 5 established habit-breaking strategies for reducing implicit bias.^58^ Third, and related to the second explanation, the core goal of cancer genetic counseling is to help individuals understand hereditary cancer risk and support informed health-related decision-making.^59,60^ Consequently, sessions elicit and prioritize patients’ unique personal and family context.^61^ These unique features of cancer genetic counseling collectively may buffer the consequences of implicit prejudice among GCs. Finally, the small sample of 15 GCs may have limited the ability to detect associations with implicit prejudice.

Among Black patients, only explicit medical-context stereotypes were associated with less-positive patient-centered outcomes. This is the first study, to our knowledge, to demonstrate that explicitly endorsed medical-context stereotypes among GCs (or any health care clinician) are associated with patient-centered outcomes, rather than being limited to clinical recommendations. These findings extend prior research on clinician bias and health care disparities^25,62^ by suggesting that explicit stereotypes may shape not only clinical recommendations but also both the content of information exchange and the interpersonal dynamics of genetic counseling sessions.

One potential mechanism is how information is framed, prioritized, and conveyed during counseling sessions. For example, GCs who strongly endorse beliefs that Black patients are more likely to be mistrustful of genetic counseling may alter their communication in ways that unintentionally create imbalance, either by overemphasizing the benefits of genetic testing while downplaying uncertainty and risks, or by overcompensating and disproportionately focusing on uncertainty and potential drawbacks. In either case, such imbalances in information delivery could be reflected in decisional conflict, which may, in turn, contribute to lower patient satisfaction. Beyond decision-related communication, explicit medical-context stereotyping may also manifest in GCs’ general interpersonal style. For instance, endorsing beliefs that Black patients are less medically cooperative may lead to more directive or paternalistic communication patterns when interacting with Black (vs White) patients. These differences in communication style may be reflected in perceived therapeutic alliance. Future research should investigate these potential underlying mechanisms.

The present study advances literature on clinician bias and health care disparities by highlighting the distinct and potentially consequential role of explicitly endorsed medical-context stereotypes, beyond the implicit bias emphasized in prior research. Clinically, the current findings suggest that current genetic counseling training may be effective in mitigating some of the well-documented effects of implicit prejudice in clinical interactions; however, it appears insufficient to fully eliminate racial disparities in patient-centered outcomes. For Black patients, repeated experiences of being perceived through the lens of negative stereotypes may contribute to mistrust of the health care system and reduced engagement with health care, and ultimately inequitable health status. Thus, understanding and addressing mechanisms underlying the association between medical-context stereotypes and patient-centered outcomes is important not only for improving individual clinician-patient interactions but also for reducing persistent racial health care disparities.

The current findings have further implications for genetic counseling education and training. Specifically, existing training likely strengthens core competencies outlined in American Society of Clinical Oncology guidelines, such as rapport-building, empathy, and patient-centered communication,^20^ which may also buffer the consequences of implicit prejudice. However, it may be important to further augment training with approaches that directly address explicit medical-context stereotypes. For example, one approach could be to help trainees critically examine how these beliefs are inaccurate and replace them with accurate information. In some instances, stereotypical beliefs may reflect partially accurate group-level patterns (eg, perceptions that Black patients may express greater mistrust of the health care system^63,64^). In such cases, training should not simply aim to correct the belief, but rather to contextualize it by addressing the historical, cultural, and structural factors that contribute to these patterns.^65^ In addition, training should equip GCs with concrete communication strategies to ensure that such expectations are not translated into differential communication behaviors. Finally, this last point reinforces an important direction for future research discussed earlier—the need to empirically examine the mechanisms through which explicit medical-context stereotypes influence patient-centered outcomes, including communication processes and interactional dynamics within genetic counseling encounters.

### Limitations

This study has limitations that should be mentioned. Although the primary aim of the present study was to examine associations between multiple forms of bias among GCs and patient-centered outcomes, it is also important to acknowledge that Black patients reported worse outcomes on most assessed dimensions despite minimal evidence of GC bias on average. The limited evidence of overall GC bias may be due to the small sample size. Indeed, a previous study found that a sample of over 200 certified GCs and trainees, on average, exhibited both implicit prejudice and implicit stereotypes.^34^ However, as noted earlier, there was substantial variability in each form of bias across GCs in the current sample.

The observed pattern of findings may also be explained by factors beyond the assessed GC biases. One such factor may be racial concordance between patients and GCs, which has been linked to perceived shared understanding, trust, and communication quality in prior research.^66,67,68,69^ Because the majority of GCs in the current sample identified as White, we were unable to isolate the effects of racial concordance. This limitation reflects the broader lack of racial diversity within the genetic counseling workforce; notably, only approximately 2% of certified GCs in the US identify as Black.^70,71^ Accordingly, future research should simultaneously investigate how GC bias and racial concordance contribute to patient-centered outcomes, particularly in larger and more diverse samples across multiple sites.

Additionally, the generalizability of our findings across genetic counseling settings may be limited because the study was conducted exclusively in cancer genetic counseling. In particular, findings related to decisional conflict may differ in other genetic counseling contexts, such as prenatal, pediatric, or cardiovascular genetic counseling, where patients’ concerns, knowledge, and decision-making needs may differ.

## Conclusions

In this cohort study of patient-clinician communication during cancer genetic counseling that focused on assessment of and counseling for hereditary cancer risk, stronger endorsement of medical-context stereotypes favoring White over Black patients was significantly associated with multiple patient-centered outcomes. These findings suggest that addressing health care disparities in genetic counseling may require moving beyond an exclusive focus on implicit prejudice to also consider explicit stereotypical beliefs and the ways they shape clinical communication. Ultimately, a more comprehensive understanding of patient-GC communication will be essential for developing interventions that meaningfully improve equity in genetic counseling care and patient-centered outcomes.

## Notes

Supplement 1. eFigure 1. Materials Visual Representation of Our Hypotheses eAppendix 1. Enrolled Participants eFigure 2. Patient Enrollment Flow Diagram eAppendix 2. Screening and Recruitment Procedure in the Parent Study

Supplement 2. Data Sharing Statement
