# Supplementary material for: Clinician Bias and Patient Experience in Cancer Genetic Counseling
Source: JAMA Netw Open. 2026 Oct 26;9(10):e2639990. doi: 10.1001/jamanetworkopen.2026.39990 (PMC13711139; doi:10.1001/jamanetworkopen.2026.39990)
Supplement: Supplementary file 1 [file jamanetwopen-e2639990-s001.pdf]

Supplemental Online Content

Hagiwara N, Shin Y, Yeasmins S, et al. Clinician bias and patient experience in cancer genetic counseling. *JAMA Netw Open*. 2026;9(10):e2639990.
doi:10.1001/jamanetworkopen.2026.39990

eFigure 1. Materials Visual Representation of Our Hypotheses

eAppendix 1. Enrolled Participants

eFigure 2. Patient Enrollment Flow Diagram

eAppendix 2. Screening and Recruitment Procedure in the Parent Study

This supplemental material has been provided by the authors to give readers additional information about their work.

© 2026 Hagiwara N et al. *JAMA Network Open*.

eFigure 1. Materials Visual Representation of Our Hypotheses

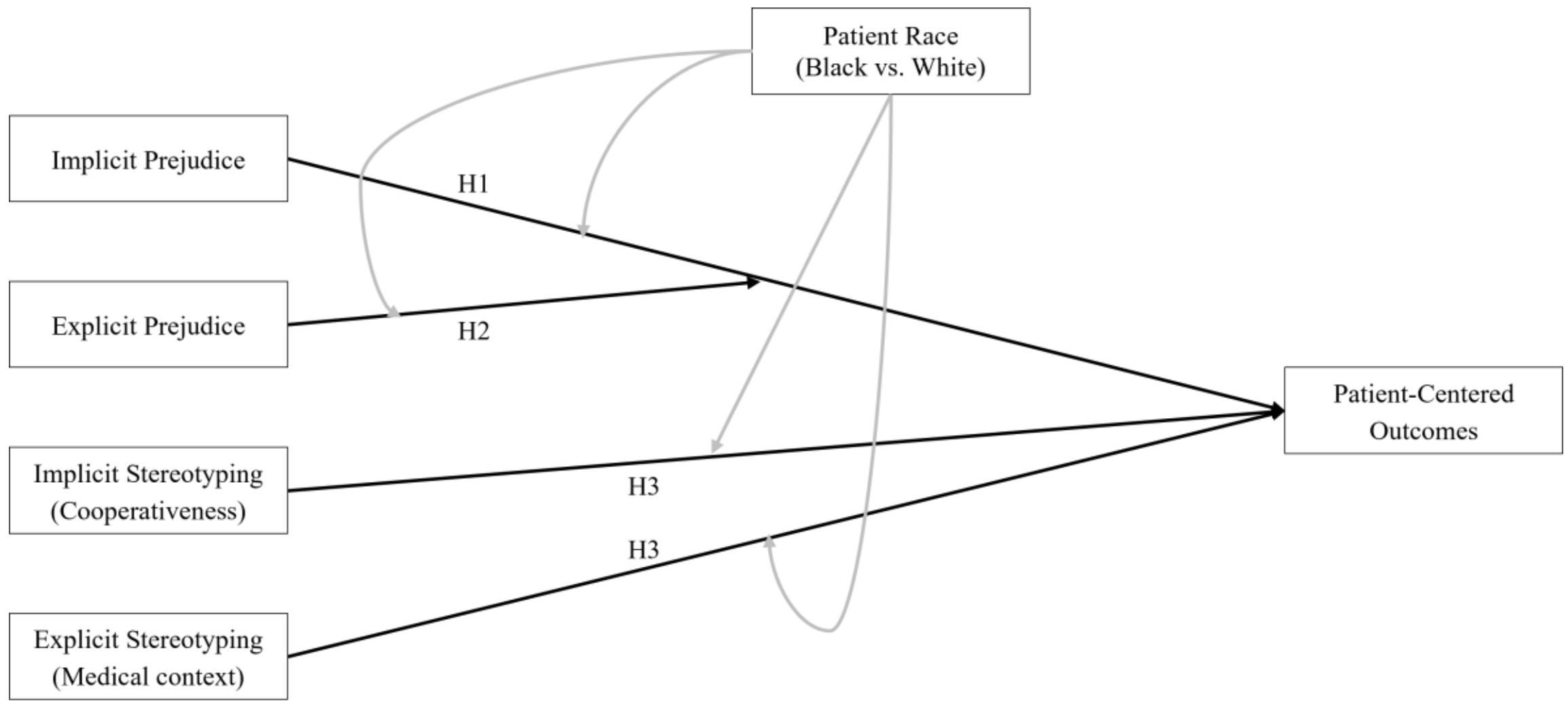

Implicit prejudice = Negative affective evaluations or feelings toward a social group and its members that are automatically and spontaneously activated, sometimes without conscious awareness or deliberate intention.

Explicit prejudice = Negative affective evaluations or feelings toward a social group and its members that individuals are consciously aware of and can deliberately report or endorse.

Implicit stereotyping = Automatically and spontaneously activated beliefs or expectations about the characteristics, behaviors, or attributes of a social group and its members. In the current study, implicit stereotyping was assessed specifically in terms of beliefs about the medical cooperativeness of Black and White individuals.

Explicit stereotyping = Consciously accessible beliefs or expectations about the characteristics, behaviors, or attributes of a social group and its members that individuals can deliberately report or endorse. In the current study, explicit stereotyping was assessed both in terms of general social stereotypes and stereotypes specific to the medical context.

H1: Higher (vs. lower) levels of implicit prejudice among GCs would be associated with less positive reactions from Black patients but more positive reactions from White patients.

H2: GCs characterized by an aversive racism profile (i.e., higher levels of implicit prejudice but lower levels of explicit prejudice) would elicit the least positive reactions from Black patients, relative to other bias profiles (i.e., high implicit prejudice and high explicit prejudice, low implicit prejudice and low explicit prejudice), but not from White patients.

H3: Stronger (vs. weaker) endorsement of the stereotype specifically related to the medical context, favoring White individuals over Black individuals, would be associated with less positive reactions from Black patients and more positive reactions from White patients.

© 2026 Hagiwara N et al. *JAMA Network Open*.

eAppendix 1. Enrolled Participants

Of 18 genetic counseling providers (8 certified genetic counselors, 10 trainees), 15 agreed to participate in the study (8 certified genetic counselors, 7 trainees). Reasons for declining to participate in the study include being too busy (*n* = 1), wanting to focus on skill set development (*n* = 1), and not being comfortable with sessions being recorded (*n* = 1). Detailed information on the number of patients at each stage of the recruitment process, along with the reasons for non-participation at each stage, is provided in the patient enrollment flow diagram below.

eFigure 2. Patient Enrollment Flow Diagram

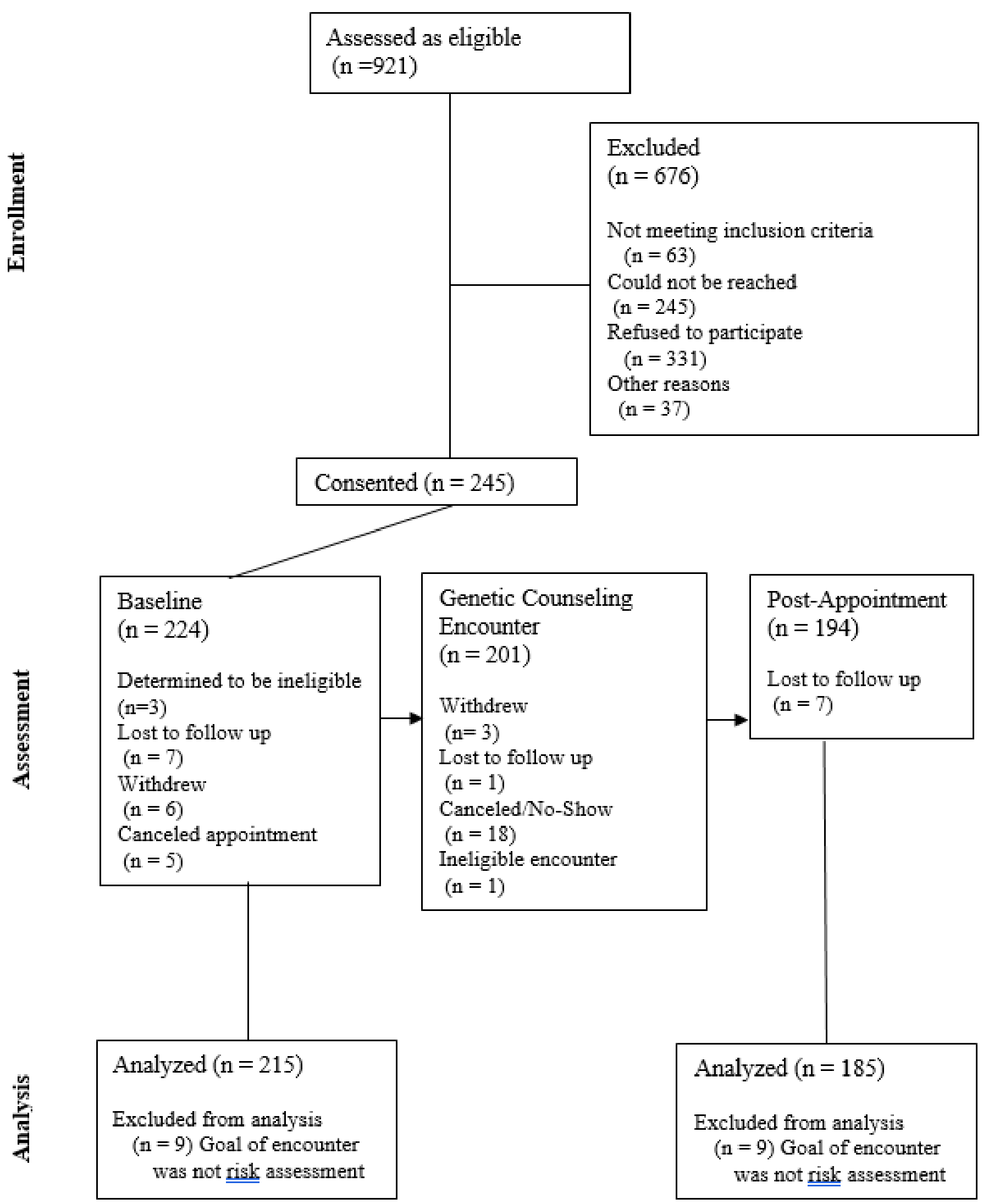

© 2026 Hagiwara N et al. *JAMA Network Open*.

eAppendix 2. Screening and Recruitment Procedure in the Parent Study

The eligibility criteria for genetic counseling providers are that they are either American Board of Genetic Counseling-certified genetic counselors or trainees in at least the second rotation for cancer genetic counseling. We approached all eligible providers in either a small group or individually to accommodate variability in their preference and scheduling.

The eligibility criteria for patients are: 1) self-identify as either Black/African American or White American; 2) ≥ 18 years old; 3) have an upcoming cancer genetic counseling appointment primarily focused on assessment of, and/or counseling for, a hereditary cancer condition with one of the participating providers at any of the recruitment sites; and 4) be able to comprehend all documents in English, written at a 6th grade reading level. Patient recruitment was conducted in sequential steps. First, we identified potentially eligible patients based on information documented in the electronic medical record. Next, we contacted these patients and assessed their eligibility based on their self-reported responses to the eligibility criteria. Finally, eligibility was confirmed based on self-reported race and age in the baseline survey.

A Monte Carlo simulation based on 5,000 simulated datasets indicated that a total sample of 220 patients would provide 80% power to detect a small-to-moderate effect (Cohen's d = 0.20) of providers' implicit racial prejudice on provider communication styles, while accounting for 10 provider-level factors and 5 patient-level factors in the model. The number of providers was not specified in this simulation because the model did not account for the nesting of patients within providers. The sample size of 15 providers was determined through a subsequent Monte Carlo simulation that did not account for patient clustering within providers.

© 2026 Hagiwara N et al. *JAMA Network Open*.
