# Supplementary material for: Clinician Bias and Patient Experience in Cancer Genetic Counseling
Source: JAMA Netw Open. 2026 Oct 26;9(10):e2639990. doi: 10.1001/jamanetworkopen.2026.39990 (PMC13711139; doi:10.1001/jamanetworkopen.2026.39990)
Supplement: Supplementary file 2 [file jamanetwopen-e2639990-s002.pdf]

Data Sharing Statement

Hagiwara. Clinician Bias and Patient Experience in Cancer Genetic Counseling. *JAMA Netw Open*. Published October 23, 2026. doi:10.1001/jamanetworkopen.2026.39990

Data
Data available: No

Additional Information
Explanation for why data not available: Our study was a secondary analysis of data from a larger study, and the parent study is still ongoing. Once the analyses of the parent data are complete, the deidentified survey data will be made available.
